# Determining multidimensional influences of network heterogeneity on university students’ psychological and academic well-being: The mediating role of social network exhaustion

**DOI:** 10.1016/j.heliyon.2024.e32328

**Published:** 2024-06-03

**Authors:** Hua Pang, Kaige Zhang

**Affiliations:** School of New Media and Communication, Tianjin University, Tianjin, 300072, China

**Keywords:** Mobile social media, Social network exhaustion, Network heterogeneity, Academic well-being, Psychological well-being, University students

## Abstract

Mobile social media has become indispensable to university students' communication with various socio-demographic populations, exposing them to diverse social networks and augmenting their network heterogeneity. Although the psychological ramifications of network heterogeneity have been extensively examined, its correlated academic outcomes remain inconclusive. The current study formulated an integrated theoretical research model based on the stressor-stress-outcome framework to investigate the influence of factors associated with network heterogeneity (specifically, privacy invasion, social comparison, self-presentation, and excessive WeChat use) on social media exhaustion, psychological well-being, and academic well-being among university students. Furthermore, the research examined the mediating effect of social network exhaustion among network heterogeneity, psychological well-being, and academic well-being. A cross-sectional survey of 1128 WeChat users revealed that social comparison and excessive WeChat use had positive associations with social network exhaustion, while privacy invasion and self-presentation were negatively correlated with social network exhaustion. Additionally, social network exhaustion was negatively correlated with psychological well-being and academic well-being. Furthermore, social network exhaustion mediated the influences of network heterogeneity on psychological well-being and academic well-being. These obtained results could contribute to a more nuanced understanding of the underlying causes of social network exhaustion and the multifaceted effects of network heterogeneity. These insights may prove valuable for practitioners to enhance university students’ psychological states and academic performance in the contemporary mobile media-saturated environment.

## 1 Introduction

In recent decades, mobile social media has been pervasively adopted, which has radically reshaped every aspect of work and life especially social connection and information sharing [[1], [2], [3]]. Owing to distinctive affordance and flexibility, the implementation of mobile social networking sites (SNS) enables users to craft virtual personas, exchange brief videos and photographs, and promptly establish connections with their real-world acquaintances [4,5]. As a result, mobile SNS platforms and Internet communities have emerged as brand-new cyber places that facilitate individuals to inhabit a digital domain. Simultaneously, a wide range of individuals who are physically unknown to each other engage in virtual communication that breaks down geographical and socioeconomic barriers. This exposure of users to diverse relationships and communication could enhance their network heterogeneity [6,7]. In Mainland China, WeChat is the most significant and commonly utilized mobile social media, with more than a billion active users per month [8,9]. Users could acquire speedy texting, mobile paying, and other types of tiny but vital applications provided by WeChat to facilitate social contact [10,11]. Essentially, WeChat has amassed an enormous number of users acquainted with each other in reality, making it possible for users to make new connections who are geographically proximate, enabling them to combine their Internet and reality social interactions via this platform [[12], [13], [14]]. As demonstrated by Dabija, Bratianu, Dominici and Vătămănescu [15] and Lăzăroiu [16], this digitalization process has precipitated the emergence of diverse manifestations of collective intelligence and novel mechanisms for peer-driven knowledge dissemination. Thus, university students have become prominent users of these ICTs such as WeChat to satisfy study and daily life demands, highlighting that mobile SNS has evolved into a crucial component for the young demographic, typically ranging from 18 to 33 years of age as shown by prior investigations [17,18].

Nonetheless, the correlation between mobile SNS use and individual well-being is intricate and multifaceted [2,19]. The current body of research has addressed the effects brought about by mobile SNS use on subjective well-being and has uncovered conflicting results [20,21]. Some investigations have revealed a variety of beneficial effects caused by mobile SNS, including the facilitation of knowledge transmission and innovation, as well as the enhancement of civic participation and social capital [7,10]. Zhou and Zhang [21] has demonstrated that using mobile SNS to maintain connections with social groups which imbue individuals with a sense of purpose and belonging tends to confer self-esteem and perceived worth. Specifically, engaging in posting activities on mobile SNS represents a swift and cost-efficient method to solicit support, which facilitates prompt and substantial aid provision, thereby potentially bolstering individuals' overall well-being [22]. Conversely, mobile SNS use may have detrimental impacts on general well-being by inducing adverse internal outcomes, such as encroachment on personal life and mental occupation [23,24], isolation sensation and diminished self-esteem [25]. As recently expounded by Eitan and Gazit [19], the enhancement of well-being does not originate from social media usage behavior, but rather from the sense of liberation and tranquility experienced upon disengagement from social media platforms, which highlights the intricate interplay between social media usage and individual well-being. These divergent results might demonstrate the necessity for additional conceptualized quantitative research to thoroughly examine the mental consequences of mobile SNS use in the contemporary digital era. Moreover, within the educational domain, numerous studies have revealed the decline in academic achievement, increased distraction, and alterations in habitual behavior due to irrational mobile SNS adoption [2,3,[26], [27], [28]]. However, limited existing research has concentrated on academic well-being, which is regarded as an inextricable factor associated with academic success [29]. Therefore, meticulously evaluating both academic well-being and psychological well-being is crucial in comprehending the influence of mobile SNS utilization on university students’ educational attainment and mental state.

Additionally, studies have demonstrated that it is critical to identify potential mediating and moderating factors, rather than the straight connection between mobile SNS implementation and subjective well-being [21,30]. For instance, Zhou and Zhang [21] have identified envy and fatigue simultaneously function a negative mediating effect between WeChat use and subjective well-being. Moreover, Wang, Wong, Wang, Li, Kim and Lee [30] has confirmed SNS addiction mediates the impact of SNS usage on both psychosocial and mental health status. Specifically, Sheng, Yang, Han and Jou [13] have indicated the significance of social network exhaustion in mediating the influences of external stimuli on users' emotional and mental states. Thus, social network exhaustion could assist in interpreting the complex links between individuals' mobile SNS use, academic well-being, and psychological well-being. With the lifestyles of individuals intensively depending on technology, more incentives have been evoked for excessive mobile SNS use. Such a situation could elicit social network exhaustion as a detrimental consequence experienced by university students [12,31]. Furthermore, as demonstrated by Andreassen [31], users with mobile SNS addiction or excessive use initially show a discernible aim of asserting control over their digital social environment, but paradoxically they find themselves relinquishing control as they become increasingly subjugated by the pervasive influence of their interconnected social networks. Wang [12] has also evidenced multiple antecedents of university students’ mobile SNS addiction, including use intensity, psychological enhancement, playfulness, and social identity. Prior literature has investigated a range of causes that contribute to social network exhaustion, which could be classified into social media-related factors and user-related factors [13,32]. As demonstrated by Sheng, Yang, Han and Jou [13], social media-related factors manifest in the form of communication overload and information oversharing attributable to social networking software or platforms, alongside the excessive utilization of system features. Besides, according to Bright, Kleiser and Grau [32], users-related factors encompass confidence, self-efficacy, privacy concerns and helpfulness, which could facilitate or impede adverse aspects of social media. Furthermore, research has revealed that social network exhaustion may engender a plethora of negative impacts, such as decreased efficiency, elevated workload and diminished usage intention [21,33]. However, despite the emergence of social network exhaustion as an alarming societal problem, its mediating function still exists uncertain and necessitates further examination [[34], [35], [36]]. By conducting mediating testing, the current study can elucidate the complex path through which social network exhaustion operates in influencing the relationship network heterogeneity and academic well-being, as well as psychological well-being.

To fill the potential research lacuna, the current study employed the stressor-strain-outcome (SSO) paradigm to thoroughly assess ramifications triggered by privacy invasion, social comparison, self-presentation, and excessive WeChat use, which could reflect individual variations in network heterogeneity [6]. The current investigation was also conducted on how these variables impacted social network exhaustion and consequently affected psychological and academic well-being, and whether social network exhaustion could mediate the effects of network heterogeneity on university students' psychological well-being and academic well-being. Moreover, Skogen, Hjetland, Bøe, Hella and Knudsen [20] have proposed that mobile SNS usage needs to be viewed as multifaceted, with individuals’ diverse behaviors associated with differential negative consequences. By delving into social media-related and user-related factors such as privacy invasion social comparison, self-presentation and excessive WeChat use, the current work complemented the emphasis of previous studies on additional underlying stressors. Furthermore, incorporating both the psychological and academic well-being of university students into an integrated study, the current work explicitly extended the studies solely focused on academic or psychological ramifications. Thereby, the results of the current research could potentially bolster the recognition of novel functions provided by recently emerging mobile SNS platforms such as WeChat, enhancing deep comprehension of the complicated impacts of mobile SNS on behaviors and internal consequences within the contemporary mobile media-saturated environment.

## 2 Literature review and hypotheses establishment

### 2.1 The fundament theoretical framework

The stressor-strain-outcome (SSO) model, which has been effectually employed in prior studies, furnishes a framework for stress and fatigue research [8,28,37,38]. This model has garnered substantial traction within the realm of Information Systems (IS) research, particularly in concert with investigations into social media utilization. In a recent investigation conducted by Li, Jiang, Yan and Li [37], the SSO model serves as the framework to elucidate correlations between social media overload, social media fatigue, anxiety and health self-efficacy. Employing the SSO model, an investigation encompassing 677 college students has underscored technostress and Internet addiction as significant antecedents of diminished quality of life [38]. In the SSO model, stressors are defined as personal and environmental stimuli that trigger stress [23]. Strains and outcomes reflect exhaustion and fatigue, producing an impact on concentration and emotion. Furthermore, the potential impact of perceived stressors on outcomes is mediated by strain [34]. Prior studies have partially substantiated the outcomes of stress-related or fatigue phenomena in the workplace such as compromised attention and reduced teamwork [8,31]. In recent years, numerous researchers have started investigating exhaustion and technostress associated with online education through the SSO model [2,28]. However, the SSO model has not been thoroughly utilized to probe academic and psychological well-being in relation to mobile SNS usage. The interactive affordance of mobile SNS allows users to establish and maintain social connections by combining user-generated content with social networking. In particular, mobile SNS provides various functions such as instant messaging, real-time notifications, and versatile platforms, which facilitate users' engagement in self-presentation. It results in university students’ exposure to extravagant information, inordinate contact, and excessive requests for social interaction. For instance, university students utilizing social media platforms may find themselves required to concurrently manage numerous communication requests, which engenders recurrent interruptions to their ongoing task at hand [37]. To stay abreast of the continuous flow of information, university students often experience a persistent pressure to remain connected with others on social networks, seeking to alleviate the tension arising from non-participation [19]. Nevertheless, in instances where the demands of usage surpass their processing capacity, university students may experience a sensation of being overwhelmed and lose control over their circumstances, leading to mobile SNS overload [8]. These disruptive conditions could impair the psychological well-being of university students and adversely affect their academic performance [29,39].

The current study selects privacy invasion, social comparison, self-presentation, and excessive WeChat use as stressors to uncover the negative aspects of mobile SNS. Privacy invasion is involuntary exposure of private space, which could heighten users' perception of risks about the security of personally identifiable information shared online [23,40]. Social comparison describes the procedure of comparing oneself to others by seeking information and evaluating oneself [41]. Additionally, self-presentation refers to the cognitive and behavioral strategies employed by individuals with the intention of constructing an optimal impression [42]. Drawing on these theoretical insights, social network exhaustion is adopted as strain. Social network exhaustion reflects an individual's mental response to the stressful situation triggered by mobile SNS usage. It highlights a subjective feeling of fatigue when individuals confront with enormous information through limited cognitive resources [33,34]. From the perspective of social network exhaustion, the utilization of mobile SNS could engender undesirable effects. The academic performance of students could be adversely affected due to insufficient time and effort allocated to study, and psychological conditions could be impacted by stressful circumstances arising from the adoption of mobile SNS in a campus environment [2,8]. Hence, the stressor-strain-outcome model is an efficacious technique for linking specific stressors (privacy invasion, social comparison, self-presentation, and excessive WeChat use), strain (social network exhaustion), and outcomes (psychological well-being and academic well-being). Consequently, the multifaceted aspects of mobile SNS implementation and network heterogeneity could be accentuated in their repercussions on university students' academic situation and mental condition.

### 2.2 Linking network heterogeneity to social network exhaustion

Network heterogeneity could shape social setting diversity and expose individuals to heterogeneous groups [1,7]. Dhar and Bose [1] have illustrated that mobile SNS afford users the ability to sustain connections with both friends and acquaintances. Consequently, users exhibit a greater degree of connectivity within mobile SNS in contrast to offline social networks. The expanded avenues for social interaction serve as a motivating factor driving users' continued engagement with SNS platforms. Furthermore, Kim and Kim [7] have initiated an inquiry into the potential positive association between social media utilization and individuals' network heterogeneity, which has furnished compelling evidence endorsing the significant role of social media engagement in facilitating interaction or communication with heterogeneous individuals. Specifically, users who engage in online activities that promote interaction with diverse individuals, such as exchanging personal information and ideas, are more likely to possess heterogeneous networks on mobile SNS [10]. Previous research has discovered that network heterogeneity enhances individual online social capital and alleviates exhaustion, culminating in favorable effects on social confidence, citizen participation, and life fulfillment [1,7]. Nevertheless, network heterogeneity can also produce detrimental ramifications. The incessant contact and information influx facilitated by the network heterogeneity of mobile SNS might induce overload, diminishing individuals’ intellectual processing ability [6]. Additionally, network heterogeneity could expose individuals to biased information and bring about cognitive dissonance [1]. Notwithstanding, extant studies have indicated that there is ample evidence supporting the salutary effects of network heterogeneity, while the deleterious effects necessitate further investigation [6,7]. Therefore, the present article extracted privacy invasion, social comparison, self-presentation, and excessive WeChat use as stressors associated with the underlying effects of network heterogeneity to ascertain whether they could be linked with social network exhaustion.

Privacy invasion happens when users are incapable of restraining or discerning access to personal data [13,43]. Mobile SNS retain and accumulate copious amounts of private information pertaining to location and social relationships, which evokes concern among users regarding their privacy [32]. Although researchers have studied the relationship between privacy invasion and exhaustion, whether privacy invasion can cause exhaustion remains controversial [13,44]. The potential threat of personal information invasion may compel users to invest supplementary efforts to familiarize themselves with privacy agreements, and the resulting cost of attention could lead to emotional exhaustion [13]. However, the opposite view is that willingness to obtain information could be weakened in situations of damage or threat, engendering discontinuous use intention [45,46]. As a result, mobile SNS users who suffered low privacy invasion may be more reachable and susceptible to information overload. Furthermore, the degree of users’ control over privacy settings significantly affects whether they experience exhaustion [47]. Users who are experienced with privacy invasion, are more inclined to recognize privacy-related contexts and implement measures to preclude subsequent invasion, decreasing the probability of experiencing exhaustion [43,44].

Social comparison occurs when individuals are devoid of objective criteria to estimate themselves and tend to compare themselves with comparable people [41,48]. Prior researchers have assessed the relationship between social comparison and exhaustion and found a positive linkage. For instance, social comparison can lead to emotional exhaustion and negative psychological states, such as envy and despondency [20]. The diffusion of the Internet facilitates the creation of self-image and simplifies the procedure of social comparison [47]. Moreover, mobile SNS provides novel approaches for individuals to observe others and accelerates the process of social comparison by incessantly providing information about others [41]. On mobile SNS, the content disseminated by individuals often highlights superior factors such as wealth and appearance to create a desirable impression. Consequently, users are inclined to engage in social comparison by juxtaposing the information of others with their own experiences and circumstances. Empirical evidence has demonstrated social comparison could detrimentally impact individuals’ well-being and could trigger diminished life satisfaction, depression, and compromised self-confidence [47,48]. Furthermore, social comparison has been identified as aggravating information overload and precipitating exhaustion [49]. After engaging in social comparison on mobile SNS, university students will encounter negative stimuli such as a sense of alienation and demotivation, which can be manifest as exhaustion [2].

Self-presentation can be considered as the act of portraying oneself favorably and managing the impressions of others by manipulating one's social background and appearance [20,42]. Several studies has suggested self-presentation has the potential to engender social network exhaustion [20,50]. Specifically, mobile SNS creates a platform for selective self-presentation through editable personal profiles and comments. Consequently, online self-presentation motivates individuals to remain attentive to social cues in order to adapt to the requirements of social contexts [47]. Nevertheless, when users discern a dissonance between the portrayed image and their authentic self, they may experience the sensation of exhaustion [50]. This situation could trigger role conflicts, causing the inability to effectively manage the demands of multiple roles [51]. Additionally, it is noteworthy that self-presentation could initiate online harassment and cyberbullying [20]. Ultimately, self-presentation generates social network exhaustion and reduces users' cognitive ability [6]. Thus, we hypothesized as follows:

H1 Privacy invasion is negatively correlated with social network exhaustion.

H2 Social comparison is positively correlated with social network exhaustion.

H3 Self-presentation is positively correlated with social network exhaustion.

### 2.3 Linking excessive WeChat use to social network exhaustion

Recently, the widespread availability of smartphones and the implementation of social media platforms have sparked discussions on excessive mobile SNS use, which describes the phenomenon of spending inordinate time and generating dependence on these platforms [27,52]. Several studies have revealed the causes and motivations of excessive mobile SNS use [12,53]. For instance, the multifunctionality and portability of mobile SNS facilitate problematic and unconscious usage among individuals [31]. Users who possess lower levels of self-control are at an increased risk of succumbing to excessive mobile SNS usage [53]. Besides, the adoption of mobile SNS is often driven by information-seeking and identity construction, leading to dependence on interpersonal relationships. The social enhancement and social support obtained from these interpersonal communications could attribute to excessive mobile SNS use [12,31].

Moreover, some scholars have interpreted excessive mobile SNS use as addictive behaviors, exhibiting compulsive concern or over-focus on mobile SNS [17,54]. When users attempt to withdraw from excessive mobile SNS use, they may experience self-blame, uneasiness, and helplessness [31]. Additionally, plenty of physical and psychological damage may be brought about by excessive mobile SNS use, such as frustration, heightened stress levels, and social isolation [30]. Furthermore, excessive mobile SNS use has been described as a form of cyber relationship addiction, featuring the fixation with online interaction relationships [54]. Based on these studies, excessive mobile SNS use can eventually be considered a behavioral addiction similar to Internet addiction, which is characterized by inordinate usage and uncontrolled concentration [5,27].

Regarding the correlation between excessive mobile SNS use and exhaustion, extant research has revealed a linkage between excessive mobile SNS use and negative emotions, including feelings of isolation and dissatisfaction [39,41]. Moreover, excessive mobile SNS use has been confirmed with cognitive preoccupation, information overload, and negative influences on individual well-being [30,55]. Consequently, it will cause an inability to regulate time spent on mobile SNS and increased fatigue [54]. With its large user base and high-frequency access, WeChat features mobility and the combination of strong and weak ties. Empirical studies have found that excessive WeChat use could engender social relationship conflicts and reduce concentration, leading to sleep disorders, anxiety, and behavior problems [5,12,17]. When excessive WeChat use occurs among college students, it induces physical or mental harm, diminished academic performance, and generates social network exhaustion [21]. Thus, we hypothesized as follows:

H4 Excessive WeChat use is positively correlated with social network exhaustion.

### 2.4 Linking social network exhaustion to psychological and academic well-being

Psychological well-being is defined as perceptions of optimal life experiences, active emotional states, and minimal levels of negative mood [39,56,57]. Prior research has identified social network exhaustion as a deleterious behavioral problem, which is manifested by diminished levels of engagement, inspiration, or vitality of online activities, but few have concentrated on the correlation between social network exhaustion and mental state [55,58]. For instance, studies have examined the external outcomes of social network exhaustion, which contains social network discontinuance, problematic smartphone use, and switching to alternative social media platforms [34,58]. Meanwhile, internal side repercussions may manifest when experiencing social network exhaustion, particularly in compromised life satisfaction and efficiency [5]. Besides, social network exhaustion manifests as unregulated social media consumption and irrational procrastination, leading to diminished self-efficacy and the neglect of familial and occupational duties [49]. Empirical evidence from Facebook users with different occupational backgrounds has corroborated that social network exhaustion could impede users’ incapacity to fulfill life and work obligations [33]. When users fail to accomplish these social responsibilities, it could induce insufficient self-esteem and ultimately compromise psychological well-being [35].

Academic well-being is intrinsically linked to educational environments and encompasses metrics such as scholastic achievement, self-worth, and stress levels [26,29]. Considering the significance of utilizing social media for online education among university students, it is vital to demonstrate the correlation between social network exhaustion and academic well-being [59]. Several studies have investigated how social network exhaustion could negatively impact academic performance, as well as how it affects university students’ cognitive states [2,47]. A recent investigation conducted by Jabeen, Tandon, Azad, Islam and Pereira [2] has confirmed that university students afflicted with addiction to social media platforms are prone to engendering an excessive preoccupation with sustaining uninterrupted connectivity to these platforms rather than concentrate on their academic objectives. Another research undertaken by Malik, Dhir, Kaur and Johri [47] has further substantiated the fatigue triggered by social comparison, self-disclosure, and high intensity of social media use could precipitate a decline in academic performance. Additionally, prior research has investigated the mobile SNS adoption of 505 valid samples from five universities and uncovered that cognitive distraction triggered by social network exhaustion could reduce GPA across different semesters [27]. Similarly, Feng, Wong, Wong and Hossain [26] have demonstrated that the frequency of Facebook indirectly affects academic performance, and academic distraction mediates the relationship between the implementation of Facebook and the decline in GPA. Except for reduced academic performance and distraction, social network exhaustion is demonstrated to slash self-regulation and impair the capacity to accomplish long-range objectives [2]. Specifically, when university students perceive a discrepancy between the time and attention invested in academic pursuits and their expectations, it may exacerbate mental distress including self-doubt and deficient life satisfaction, which ultimately affects academic well-being [23,29]. Eventually, we hypothesized as follows:

H5 Social network exhaustion is negatively correlated with academic well-being.

H6 Social network exhaustion is negatively correlated with psychological well-being.

## 3 Study methodologies

### 3.1 Study model

**Fig. 1:**
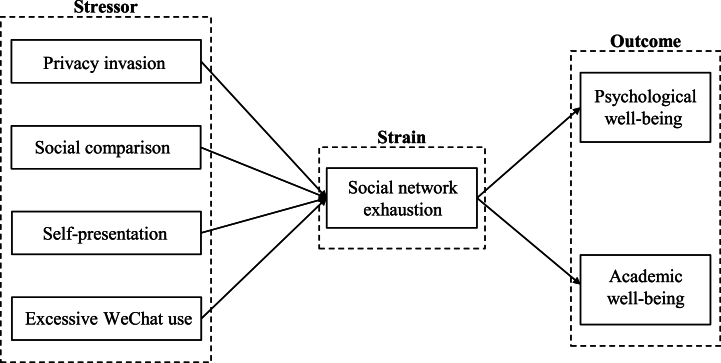
Conceptual research model.

The stressor-strain-outcome (SSO) framework was utilized by the current research to systematically examine how network heterogeneity and social network exhaustion could affect psychological and academic well-being. The current research posited that privacy invasion had a negative association with social network exhaustion, while social comparison, self-presentation, and excessive WeChat use had positive associations with social network exhaustion. Furthermore, social network exhaustion was premised to have negative correlations with psychological well-being and academic well-being. Additionally, social network exhaustion was assumed to mediate the correlations among network heterogeneity, psychological well-being, and academic well-being. Fig. 1 depicts the theoretical research model containing hypotheses and variables.

### 3.2 Participants and data collection

The present research selected its participants based on two primary criteria. First, participants were required to have experience using WeChat and to use it on a daily basis for the sake of effectiveness. Second, previous research has indicated that young generation from 18 to 33 years of age is the main user of mobile SNS [18,25,50]. Given the study's focus on the academic and psychological well-being of college students, participants were recruited from college student population spanning all academic years. Empirical data was gathered by a web-based survey administered on Sojump (www.sojump.com) between February 21 and March 16, 2023. Sojump is one of the prominent online survey platforms in China, boasting an average of over 10 million respondents completing questionnaires on a daily basis. The sampling service offered by this platform facilitates the precise distribution of questionnaires for researchers and can be conveniently shared on WeChat [36,40]. The questionnaire was distributed to several WeChat groups in mainland China and participants were invited to complete it. Considering that WeChat use is the primary focus of the study and draws upon s robustness demonstrated by previous research with similar aims conducted in China, such as investigations by Sheng, Yang, Han and Jou [13] and Niu, Yao, Tian, Sun and Zhou [49], utilizing a survey based on WeChat was well-suited for examining both academic performance and emotional states in the context of mobile SNS use. The current research conducted a pre-test involving 50 participants to ensure that the meaning of each statement was accurately conveyed without any misunderstanding. Subsequently, the current study invited two professors in Information Systems and two professors in Communication to review the questionnaire, aiming to enhance its overall quality. After this, the current research's purpose was conveyed to all participants, ensuring that their responses were based on complete voluntariness and anonymity. A total of 1160 eligible respondents participated, and after removing the suspect responses (e.g., “strongly disagree” for eight consecutive options), 1128 valid samples were retained. Table 1 presents the demographics of study samples and their general WeChat usage.

**Table 1:** Demographics of study samples and general WeChat usage (N = 1128).

Categories	Frequency	Percentage (%)
Gender		
Male	440	39.0
Female	688	61.0
Age		
18–21 years old	360	31.9
22–25 years old	480	42.6
26–29 years old	156	13.8
30–33 years old	132	11.7
Education background		
Undergraduate students	824	73.0
Graduate students	252	22.3
Doctoral students	52	4.6
Years of using WeChat		
1–2 years	12	1.1
2–3 years	60	5.3
3–4 years	124	11.0
**>**4 years	932	82.6
Frequency		
<1 time a day	8	0.7
1–2 times a day	184	16.3
2–3 times a day	232	20.6
3–4 times a day	232	20.6
**>**4 times a day	472	41.8

### 3.3 Measurement

#### 3.3.1 Questionnaire development

The founding and enhancement of the questionnaire were grounded in the extant social media literature. All the items and statements are shown in Table 2. The principal survey instrument was initially devised in the English language, subsequently undergoing the back-translation method to juxtapose the English rendition of the questionnaire with its Chinese equivalent. This method, advocated by antecedent research endeavors undertaken in China, entails a native Chinese translator translating the English statements into Chinese, followed by a native English speaker re-translating them into English, thus ensuring the preservation of the original semantic meaning [13,18].

**Table 2:** Measurements and questionnaire.

Variable	Item	Source
**Privacy Invasion (PI)**	PI1 Someone has shared my mobile social networking site account information without authorization.	Loh, Lee, Loh, Tan, Ooi and Dwivedi [44]
	PI2 Someone has used my photo on my mobile social networking account without my permission.	
	PI3 Someone has stolen my comments and opinions from my mobile social networking sites and reposted them as if they were their own.	
**Social Comparison (SC)**	SC1 I often compare myself with others with respect to what I have accomplished in life on WeChat.	Masood, Luqman, Feng and Ali [27]
	SC2 I always compare how I perform tasks to how others perform them on WeChat.	
	SC3 I am the type of person who compares often with others on WeChat.	
	SC4 If I want to find out how well I have done something, I compare what I have done with how others have done on WeChat.	
	SC5 I engage in comparing my social performance, such as social skills, popularity, and likability, in relation to others on WeChat.	
**Self-presentation (SP)**	SP1 It matters to me that my posts get a lot of likes and/or comments.	Skogen, Hjetland, Bøe, Hella and Knudsen [20]
	SP2 It is important for me to have many followers on WeChat Moments.	
	SP3 The response I get for what I post (images/status updates/stories) in WeChat Moments impacts how I feel.	
**Excessive WeChat use (EWU)**	EWU1 Using WeChat sometimes interferes with other（e.g. study and work).	Cao, Gong, Yu and Dai [4]
	EWU2 Sometimes, using WeChat has negatively impacted my social life.	
	EWU3 I may become upset occasionally when I'm not utilizing WeChat.	
	EWU4 I find it difficult to control my WeChat use.	
	EWU5 I have made unsuccessful attempts to reduce the time using WeChat.	
**Social Network Exhaustion (SNE)**	SNE1 I feel tired when using WeChat.	Pradhan [33]
	SNE2 I feel bored when using WeChat.	
	SNE3 I feel drained from using WeChat.	
	SNE4 I feel worn out from using WeChat.	
	SNE5 I feel disinterested in whether there are new things happening on WeChat.	
	SNE6 The notifications or alerts of new postings on WeChat don't really affect me.	
**Psychological Well-being (PW)**	PW1 I think I live the life I want.	Choi and Noh [56]
	PW2 I have a healthy mind.	
	PW3 My life is active and motivated.	
**Academic Well-being (AW)**	AW1 I get good grades on exams.	Deniz, Satici, Doenyas and Griffiths [29]
	AW2 I enjoy discovering something new in school.	
	AW3 I finish my assignments on time.	
	AW4 I believe that I am a good student.	
	AW5 I enjoy doing my schoolwork.	
	AW6 I have faith in my capacity to handle my academic work.	

#### 3.3.2 Privacy invasion

The article used the 3-item scale adapted from Loh, Lee, Loh, Tan, Ooi and Dwivedi [44] to measure privacy invasion. A sample item is “Someone has used my photo on my mobile social networking account without my permission”. This scale ranged from 1 (never) to 5 (always), with a Cronbach's α of 0.803 (M = 2.18, SD = 1.21).

#### 3.3.3 Social comparison

The 5-item scale adapted from Masood, Luqman, Feng and Ali [27] was utilized to measure social comparison, specifically emphasizing non-directional social comparison. Sample items contain “I always compare how I perform tasks to how others perform them on WeChat” and “I engage in comparing my social performance, such as social skills, popularity, and likability, in relation to others on WeChat”. This scale ranged from 1 (strongly disagree) to 5 (strongly agree). The Cronbach's α value was 0.859, with good reliability (M = 3.47, SD = 1.33).

#### 3.3.4 Self-presentation

The study employed a 3-item scale developed by Skogen, Hjetland, Bøe, Hella and Knudsen [20] to assess self-presentation. A representative item is “It matters to me that my posts get a lot of likes and/or comments”. Participants responded using a Likert scale ranging up to five points, with 1 for strongly disagree and 5 for strongly agree, and Cronbach's α value was 0.818 (M = 3.62, SD = 1.38).

#### 3.3.5 Excessive WeChat use

A 5-item established by a previous study was applied to evaluate excessive WeChat use [4]. Sample items are “I may become upset occasionally when I'm not utilizing WeChat” and “Sometimes, using WeChat has negatively impacted my social life”. Based on a 5-point Likert scale, students submitted their answers (1 for strongly disagree, 5 for strongly agree). The Cronbach's α value was 0.873, revealing good reliability (M = 3.01, SD = 1.71).

#### 3.3.6 Social network exhaustion

The research used a 6-item scale developed by Pradhan [33] to assess university students' social network exhaustion. The representative item is “The notifications or alerts of new postings on WeChat don't really affect me”. The measurement instrument utilized a Likert scale ranging from 1 (indicating strongly disagree) to 5 (indicating strongly agree). The internal consistency of the measure was evaluated using Cronbach's α coefficient, yielding a value of 0.928, demonstrating a high level of reliability (M = 2.94, SD = 1.72).

#### 3.3.7 Psychological well-being

The assessment of psychological well-being covered three items derived from a previous study [56]. A sample item is “I have a healthy mind”. A Likert scale with a range of 1 (strongly disagree) to 5 (strongly agree) was given to respondents to report their psychological well-being. This scale demonstrated good reliability, as evidenced by the Cronbach's α value of 0.852 (M = 3.47, SD = 1.48).

#### 3.3.8 Academic well-being

The academic well-being scale consisted of six statements modified from Deniz, Satici, Doenyas and Griffiths [29], with representative items like “I enjoy discovering something new in school” and “I have faith in my capacity to handle my academic work”. Students were presented with a Likert scale ranging from 1 (strongly disagree) to 5 (strongly agree) to assess their academic well-being. This Cronbach's α value was 0.910, indicating good reliability (M = 3.52, SD = 1.34).

## 4 Data analysis procedure

The current study employed IBM SPSS version 24.0 and IBM AMOS version 24.0 to analyze the statistical data. To start, Microsoft Excel was utilized for data cleaning to filter out inaccurate responses. Next, a test for confirmatory factor analysis and the discriminant validity of the constructs was conducted. Goodness-of-fit indices, including χ^2^/d.f, RMSEA, IFI, CFI, AGFI, and GFI, were used to evaluate model fit. Besides, the proposed hypotheses were tested by applying the structural equation modeling (SEM) procedure. Consequently, mediation analysis was conducted to determine whether social network exhaustion could mediate the effects of privacy invasion, social comparison, self-presentation, and excessive WeChat use on psychological well-being and academic well-being.

## 5 Results

### 5.1 Assessment of the measurement model

Prior researchers have recommended employing a two-step model-building approach to attain interpretable results [12,31,60]. Initially, before the path analysis, confirmatory factor analysis (CFA) was conducted to assess the overall fit of the measurement model, as well as the reliability and validity of each construct. Optimal values for model fit include RMSEA values should be below 0.08, χ^2^/d.f. values should not exceed 3, AGFI as well as GFI should be greater than 0.8, IFI and CFI should be greater than 0.9 [1]. Table 2 presents the measurement model of this study and the model fit (χ^2^/d.f. = 1.680, RMSEA = 0.049, IFI = 0.944, CFI = 0.944, AGFI = 0.833, GFI = 0.861). Besides, Table 3 outlines the statistical information about reliability and validity of the variables. All seven constructs had composite reliability (CR) values above 0.8, indicating good internal consistency. Moreover, convergent validity was assessed through the utilization of factor analysis, computing the average variance extracted (AVE), and scrutinizing squared multiple correlations (SMC) [2]. The loadings of the major reflective items exceeded 0.7, thus confirming the scale's strong convergent validity. Each AVE outweighed a threshold of 0.5, suggesting satisfactory convergence validity. Additionally, most SMC parameters were above 0.5, demonstrating convergent validity [55]. Researchers have suggested that square root of every variable's AVE value should surpass coefficients among variables to exhibit discriminant validity [13]. As shown in Table 4, every variable's square root of AVE exceeded the coefficients between the variables, confirming the discriminant validity. In conclusion, the measurement model employed in this study demonstrated a high level of model fit, satisfactory reliability, and appropriate convergent and discriminant validity.

**Table 3:** Fit indices for the measurement model and structural model.

	χ^2^/d.f.	RMSEA	IFI	CFI	AGFI	GFI
	(<3)	(<0.08)	(>0.9)	(>0.9)	（>0.8）	（>0.8）
**Measurement model**	1.680	0.049	0.944	0.944	0.833	0.861
**Structural model**	2.089	0.062	0.907	0.906	0.808	0.834

**Table 4:** Statistical outcomes of confirmatory factor analysis.

Constructs and items	Loading (>0.7)	SMC (>0.5)	CR (>0.7)	AVE (>0.5)
Privacy invasion（PI）				
PI1	0.688	0.473	0.804	0.579
PI2	0.783	0.613		
PI3	0.807	0.652		
Social comparison (SC)				
SC1	0.771	0.594	0.861	0.553
SC2	0.738	0.544		
SC3	0.682	0.465		
SC4	0.77	0.593		
SC5	0.753	0.567		
Self-presentation (SP）				
SP1	0.777	0.604	0.818	0.599
SP2	0.781	0.609		
SP3	0.764	0.584		
Excessive WeChat use（EWU）				
EWU1	0.786	0.605	0.873	0.580
EWU2	0.837	0.637		
EWU3	0.680	0.785		
EWU4	0.769	0.773		
EWU5	0.725	0.773		
Social network exhaustion（SNE）				
SNE1	0.859	0.738	0.928	0.683
SNE2	0.846	0.716		
SNE3	0.849	0.721		
SNE4	0.822	0.675		
SNE5	0.783	0.613		
SNE6	0.796	0.634		
Psychological well-being (PW)				
PW1	0.784	0.614	0.855	0.663
PW2	0.781	0.610		
PW3	0.875	0.766		
Academic well-being（AW）				
AW1	0.724	0.524	0.909	0.626
AW2	0.741	0.548		
AW3	0.839	0.703		
AW4	0.826	0.683		
AW5	0.774	0.599		
AW6	0.835	0.698		

*Notes:* SMC, squared multiple correlations; CR, construct reliability; AVE, average variance extracted.

### 5.2 Assessment of the structural model

**Fig. 2:**
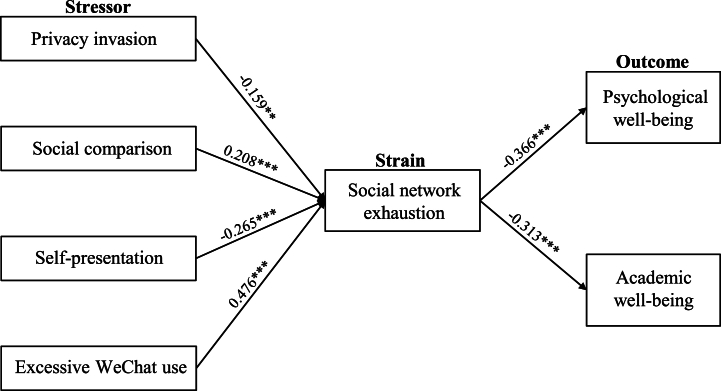
Results of structural path analysis. *Notes:* **p < 0.01, ***p < 0.001.

The present article utilized AMOS 24.0 to probe the structural model and model fit indices (χ^2^/d.f. = 2.089, RMSEA = 0.062, IFI = 0.907, CFI = 0.906, AGFI = 0.808, GFI = 0.834), demonstrating good model fit. Subsequently, the research adopted path analysis to examine the hypotheses. Table 5 displays standardized path coefficients and their significance for the hypotheses. Social comparison and excessive WeChat use were positively correlated with social network exhaustion (path coefficients of 0.208 and 0.476, p < 0.001, respectively), supporting H2 and H4. Privacy invasion (β = −0.159, p < 0.01) and self-presentation (β = −0.265, p < 0.001) exerted negative correlations with social network exhaustion, confirming H1 but rejecting H3. Furthermore, social network exhaustion was negatively correlated with both psychological well-being and academic well-being (path coefficients of −0.366 and −0.313, p < 0.001, respectively). Hence, H5 and H6 were statistically supported. Fig. 2 depicts the outcomes of the structural model.

**Table 5:** Discriminate validity-Pearson's correlation coefficient.

	PI	SC	SP	EWU	SNE	PW	AW
**PI**	0.761						
**SC**	−0.333***	**0.744**					
**SP**	0.192*	0.194**	**0.774**				
**EWU**	−0.029	0.418***	0.084	**0.762**			
**SNE**	−0.266***	0.377***	−0.206**	0.518***	**0.826**		
**PW**	0.018	−0.091	0.152*	−0.207**	−0.362***	**0.814**	
**AW**	0.024	−0.049	0.248***	−0.228**	−0.300***	0.673***	**0.791**

*Notes:* ***p < 0.001, **p < 0.01, *p < 0.05. The square root of each construct's AVE is shown as diagonal elements. The squared correlations between variables are shown as nondiagonal elements.

Following the hypothesis examination, the current study conducted mediation analysis using bootstrapping method to explore the potential mediating role of social network exhaustion between network heterogeneity, psychological well-being, and academic well-being. The indirect effect of privacy invasion on psychological well-being through social network exhaustion was significant (β = 0.049, p = 0.03 < 0.05; 95 % CI = [0.004, 0.13]), while the direct effect was not significant, thus social network exhaustion completely mediated the relationship between privacy invasion and psychological well-being. Additionally, privacy invasion had a slightly indirect effect on academic well-being (β = 0.031, p = 0.04 < 0.05; 95 % CI = [0.001, 0.094]), and the direct effect was not significant, indicating social network exhaustion played a complete mediation role between privacy invasion and academic well-being. Likewise, social comparison had an indirect influence on psychological well-being (β = −0.065, p = 0.025 < 0.05; 95 % CI = [−0.176, −0.005]), while the direct effect was not significant, indicating the relationship between social comparison and psychological well-being was completely mediated by social network exhaustion. Moreover, social comparison exhibited an indirect effect on academic well-being (β = −0.041, p = 0.039 < 0.05; 95 % CI = [−0.134, −0.001]), and the direct effect was not significant, showing social network exhaustion completely mediated the correlation between them. Besides, the indirect effect of self-presentation on psychological well-being was significant (β = 0.088, p = 0.003 < 0.05; 95 % CI = [0.03, 0.188]), and the direct effect was not significant, revealing social network exhaustion completely mediated the relationship between them. Self-presentation had an indirect effect on academic well-being (β = 0.055, p = 0.024 < 0.05; 95 % CI = [0.007, 0.14]), while the direct effect was also significant (β = 0.249, p = 0.014 < 0.05; 95 % CI = [0.054, 0.442]), demonstrating social network exhaustion partially mediated the association between self-presentation and academic well-being. Additionally, excessive WeChat use had an indirect effect on psychological well-being (β = −0.158, p = 0.003 < 0.05; 95 % CI = [−0.299, −0.063]), while the direct effect was not significant. Thus, social network exhaustion completely mediated excessive WeChat use and psychological well-being. Meanwhile, excessive WeChat use had an indirect effect on academic well-being (β = −0.099, p = 0.024 < 0.05; 95 % CI = [−0.218, −0.014]), and the direct effect was not significant, indicating that social network exhaustion played a complete mediating role between excessive WeChat use and academic well-being. Table 6 and Table 7 present the results of the mediation analysis.

**Table 6:** Statistical results of the structural model.

Hypotheses	Path	Path coefficient	p-value	Results
H1	Privacy invasion → Social network exhaustion	−0.159	0.007**	Supported
H2	Social comparison→ Social network exhaustion	0.208	0.000***	Supported
H3	Self-presentation → Social network exhaustion	−0.265	0.000***	Refused
H4	Excessive WeChat use → Social network exhaustion	0.476	0.000***	Supported
H5	Social network exhaustion → Psychological well-being	−0.366	0.000***	Supported
H6	Social network exhaustion → Academic well-being	−0.313	0.000***	Supported

**Table 7:** Analysis of the mediation effect.

Path	Effect	β	Lower	Upper	p-value	Test result
PI-SNE-PW	Indirect effect	0.049	0.004	0.130	0.030	Completely mediation
	Direct effect	−0.117	−0.323	0.083	0.257	
SC-SNE-PW	Indirect effect	−0.065	−0.176	−0.005	0.025	Completely mediation
	Direct effect	−0.008	−0.256	0.222	0.917	
SP-SNE-PW	Indirect effect	0.088	0.03	0.188	0.003	Completely mediation
	Direct effect	0.142	−0.064	0.362	0.172	
EWU-SNE-PW	Indirect effect	−0.158	−0.299	−0.063	0.003	Completely mediation
	Direct effect	−0.046	−0.289	0.167	0.661	
PI-SNE-AW	Indirect effect	0.031	0.001	0.094	0.040	Completely mediation
	Direct effect	−0.095	−0.305	0.094	0.303	
SC-SNE-AW	Indirect effect	−0.041	−0.134	−0.001	0.039	Completely mediation
	Direct effect	0.014	−0.209	0.247	0.902	
SP-SNE-AW	Indirect effect	0.055	0.007	0.14	0.024	Partially mediation
	Direct effect	0.249	0.054	0.442	0.014	
EWU-SNE-AW	Indirect effect	−0.099	−0.218	−0.014	0.024	Completely mediation
	Direct effect	−0.149	−0.389	0.061	0.187	

## 6 Discussion

### 6.1 Summary of key outcomes

The present article's primary objective was to investigate the potential impact of network heterogeneity on social network exhaustion, and its subsequent effects on psychological well-being and academic well-being. It could elucidate the ramifications of mobile SNS implementation and social network exhaustion among young generation from 18 to 33 years of age. Grounded on the SSO (stressor-strain-outcome) model, this study systematically explored the direct and indirect effects of four variables of network heterogeneity on social network exhaustion, psychological well-being, and academic well-being. The results offered substantial evidence supporting the theoretical paradigm, as well as demonstrating its reliability and validity. Besides, the findings demonstrated that privacy invasion, social comparison, self-presentation, and excessive WeChat use were significant predictors of both academic and psychological well-being.

Firstly, consistent with anticipations, it was uncovered that privacy invasion had a negative correlation with social network exhaustion, thereby, H1 was verified. Researchers have revealed that exposure to threatening situations can weaken individuals’ motivation to acquire information, and privacy invasion could engender discontinuous use intention of mobile SNS [45,46]. Besides, protective motivation theory has revealed that individuals may cultivate protective intentions in reaction to perceived threats through a process of cognitive appraisal [61]. Individuals with more privacy invasion experience would exhibit a diminished use intention. It may potentially prevent them from elevated cognitive strain, which could be significantly correlated with social network exhaustion [34]. Additionally, the current study revealed a negative correlation between self-presentation and social network exhaustion, leading to the rejection of H3. The result was inconsistent with other literature indicating a positive correlation between self-presentation and fatigue [50,51]. The reasons for this divergence may be as follows. Researchers have unpacked that social media confidence has a negative correlation with social network exhaustion [32]. Especially, self-presentation has been uncovered as a positive predictor of self-esteem, which could be beneficial to subjective well-being [25,42]. Hence, it can be deduced that individuals who actively participate in self-presentation are inclined to possess heightened confidence levels and may consequently encounter less social network exhaustion. As the sample population of this study consisted primarily of college students, particularly undergraduates, the characteristics of this group may contribute to the observed negative correlation between self-presentation and social network exhaustion.

Secondly, this study demonstrated that social comparison and excessive WeChat use were positively correlated with social network exhaustion, supporting H2 and H4. It was in line with the results released by prior investigations [47,54]. People normally opt for others who share comparable circumstances for social comparison, and mobile SNS creates avenues for them to make social comparisons with their acquaintances [48]. Users curate their online personas and engage in disseminating these contents out of the need for social comparison, which would exacerbate their social network exhaustion. Furthermore, mobile SNS provides a constant stream of information pertaining to others’ current situations, which could potentially subject individuals to excessive information and induce social network exhaustion [41,49]. Particularly, college students are susceptible to adverse outcomes such as feelings of alienation and demotivation, as a result of engaging in social comparison [2]. Prior literature has posited excessive WeChat use constitutes a manifestation of addictive behavior, which would provoke social network exhaustion. When users encounter obstacles in managing their WeChat use, it may trigger negative emotions such as frustration and anxiety [4,54]. Individuals who allocate extensive time to utilizing WeChat could experience a decrease in available time for addressing tangible issues, leading to entanglements in personal or professional disputes [31]. Additionally, succumbing to excessive WeChat usage holds the potential to induce physical problems, including sleep disturbances and decreased vitality, which can be delineated as distinct manifestations of social network exhaustion [52].

Thirdly, the current research indicated social network exhaustion was negatively correlated with psychological well-being and academic well-being, confirming H5 and H6. These outcomes were aligned with predictions and previous literature [29,35]. Social network exhaustion provokes adverse sentiments that detract from the positive experiences of life and psychological feelings. It could significantly reduce work efficiency and create irrational procrastination, resulting in impairments of the ability to manage their life and work responsibilities [5,33]. The circumstance would initiate disregard for family and daily life, evoking isolation and despondency. Consequently, these factors can emerge as significant contributors to the deterioration of psychological well-being [34]. Additionally, social network exhaustion is considered to impair students' academic capacity, particularly in their self-regulation ability. When students are exposed to social network exhaustion, they are incapable of dedicating enough time and become distracted and unfocused on their studies [23]. Not only would this affect students’ academic performance, but it could also decrease their academic confidence and self-esteem [2]. They may experience negative emotions such as apprehension and distress. Moreover, the inability to perform well academically will heighten the pressure they feel from peer competition, leading to lower self-evaluation and ultimately having a detrimental effect on academic well-being [27].

Fourthly, the study confirmed the mediating effect of social network exhaustion on the ramifications of network heterogeneity on university students' academic and psychological well-being. Precisely, the findings indicated that social network exhaustion completely mediated the impact of privacy invasion on psychological well-being as well as academic well-being. Previous academic research suggests that elevated levels of privacy intrusion experienced by users may result in decreased intentions for sustained usage, consequently manifesting in reduced occurrences of social network exhaustion [45,46]. The obtained results further substantiated this perspective, underscoring the distinctive function of social network exhaustion in dominating this process rather than the direct effect of privacy invasion. Besides, social network exhaustion was found to have a complete mediating effect on the influence of social comparison on academic well-being and psychological well-being. The results bolstered previous research, illustrating that individuals' inclinations to engage in social comparison on online platforms substantially affect their well-being, with this effect primarily mediated by the experience of social network exhaustion [24,49]. Furthermore, the findings indicated that the influence of self-presentation on psychological well-being was entirely mediated by social network exhaustion. However, concerning its impact on academic well-being, social network exhaustion served as only a partial mediator, suggesting self-presentation has a direct positive on university students' academic well-being. Building on previous studies indicating that individuals who engage in increased self-presentation may demonstrate stronger confidence and have more fulfilling lifestyles, which could potentially contribute to diminished social network exhaustion [25,42]. Hence, it can be deduced that self-presentation contributes to enhanced psychological and academic well-being through the mitigation of social network exhaustion. Considering that college students’ self-presentation content is intertwined with their academic achievement, the direct positive impact of self-presentation on academic well-being is plausible [47]. Additionally, social network exhaustion functions complete mediation role in relation to the impact of excessive WeChat use on both psychological and academic well-being. It aligns with previous research that implies the adverse consequences of excessive WeChat usage on well-being are predominantly conveyed through the phenomenon of social network exhaustion [27,36]. Finally, the findings of the study sheds light on the underlying mechanisms of social network exhaustion and facilitating the development of targeted interventions to prevent this phenomenon in a practical approach, which could foster the progress toward alleviating this societal issue.

### 6.2 Theoretical and practical implications

Some notable theoretical implications could be yielded from the present work. Firstly, despite previous investigations that have primarily unpacked the positive outcomes of online heterogeneity [1,7], its negative side has been ignored and warrants further examination [6]. By selecting four variables pertinent to the adverse effects of network heterogeneity, this study revealed that the enhanced network heterogeneity could engender university students' social network exhaustion, generating a comprehensive insight into the multifaceted nature of network heterogeneity. Secondly, prior literature has identified various antecedents as causes of social network exhaustion, such as perceived playfulness, depression, information overload, as well as mental stress [49,54]. The present findings confirmed the potential impact of privacy invasion, social comparison, self-presentation, and excessive WeChat use on social network exhaustion, which could enrich the focus on social network exhaustion and reveal additional sources of stressors. Thirdly, the current corpus of literature concerning social network exhaustion has yet to amalgamate both academic and psychological repercussions within a unified investigation, and its mediating role requires further investigation [21,47]. The current research unveiled the adverse impact of social network exhaustion on psychological and academic well-being, augmenting the ongoing research on the ramification of mobile SNS adoption among university students. Additionally, building upon the recurrent emphasis of prior studies on the mediating function of social network exhaustion [2,36], the present article delineated social network exhaustion could mediate the correlations among network heterogeneity, academic well-being, and psychological well-being. This finding elucidated that network heterogeneity does not exert a direct on university students’ academic and psychological well-being, instead, it operated through the intermediary role of social network exhaustion. Consequently, the study introduced a novel perspective aimed at thoroughly elucidating the underlying mechanisms of social network exhaustion within the framework of the SSO theoretical model.

Practically, the current study provided valuable insights for mobile SNS service providers, administrators, psychological counselors, and mobile SNS users, especially university students. Firstly, given the escalating apprehensions regarding the adverse consequences of social network exhaustion, it becomes imperative for technology companies to conduct thorough evaluations of novel functionalities that encourage or entice excessive usage of mobile SNS use. Services designers are recommended to provide users with enhanced setting options and notification tools to mitigate screen usage and login times. Secondly, in light of the noteworthy influence of excessive WeChat use, the aim of evading mobile SNS addiction involves a collaborative effort between administrators and psychological counselors. Educational institutions and counselors may incorporate additional strategies and initiatives intended for encouraging students to engage in face-to-face interactions and promoting a healthy balance between online and offline activities. Thirdly, university students should properly regulate their utilization of mobile SNS and be warned of the mechanisms and potential outcomes of social network exhaustion. As the current article revealed social comparison was positively correlated with social network exhaustion, university students may be advised to decrease reliance on social media by improving their self-control abilities. Besides, considering the negative correlation observed between self-presentation and social network exhaustion in the current research, engaging in appropriate self-presentation may boost the self-confidence and overall well-being of university students.

## 7 Conclusion, limitations and implications for future studies

The current study employed the stressor-strain-outcome (SSO) framework to systematically assess how network heterogeneity and social network exhaustion may impact psychological and academic well-being. Specifically, the research extensively investigated the mediating role of social network exhaustion to uncover the underlying mechanism, rather than focusing solely on direct influence. However, it could not be devoid of its constraints, warranting consideration in future studies. Firstly, owing to financial and space constraints, the respondents were restricted to individuals aged 18–33 from a single country. Samples from different countries may yield divergent results due to a multitude of factors. Although young people exhibit more active engagement in mobile SNS adoption, it would be prudent to include a more diverse age range to enhance the generalizability of the findings. Besides, given the unique affordances of various social media platforms, it is advisable for the upcoming study to encompass a more extensive array of platforms. Secondly, the current study examined the effects of four variables on social network exhaustion within the domain of network heterogeneity. However, social network exhaustion is influenced by a multitude of factors, and it would be advantageous for future research models to incorporate additional variables. This approach is anticipated to yield more comprehensive and nuanced insights. Thirdly, the inherent limitations of the current study stem from its cross-sectional survey design. In future investigations, employing alternative research methodologies such as longitudinal studies and focus group interviews may offer a more comprehensive and robust understanding of the relationships among these variables.

## Ethical statement and consent to participate

Approval was obtained from the ethics committee of Tianjin University with reference number: TJWHSX2302-01. Prior to data collection, written informed consent was obtained from study participants and actual data collection was conducted. The procedures used in this study adhere to the tenets of the Declaration of Helsinki.

## Funding

This research was funded by the Tianjin 10.13039/501100011788Philosophy and Social Science Planning Project (Grant No. TJWHSX2302-01).

## Data availability

The datasets used and/or analyzed during the current study are available from the corresponding author on reasonable request.

## CRediT authorship contribution statement

**Hua Pang:** Writing – review & editing, Writing – original draft, Funding acquisition, Formal analysis, Conceptualization. **Kaige Zhang:** Writing – review & editing, Writing – original draft, Methodology, Investigation, Formal analysis.

## Declaration of competing interest

The authors declare the following financial interests/personal relationships which may be considered as potential competing interests:Hua Pang is an Associate Editor for Heliyon and was not involved in the editorial review or the decision to publish this article. If there are other authors, they declare that they have no known competing financial interests or personal relationships that could have appeared to influence the work reported in this paper.
